# EEG response in humans for frequency-tagged anticorrelated random-dot stereograms: Increased coherency and alpha oscillations

**DOI:** 10.3389/fnins.2022.909225

**Published:** 2022-07-27

**Authors:** Zoltan Derzsi

**Affiliations:** ^1^Department of Psychology, New York University Abu Dhabi, Abu Dhabi, United Arab Emirates; ^2^Center for Artificial Intelligence and Robotics, New York University Abu Dhabi, Abu Dhabi, United Arab Emirates; ^3^Institute of Neuroscience, Newcastle University, Newcastle upon Tyne, United Kingdom

**Keywords:** EEG, frequency tagging, coherency, ITC, stereogram, disparity, anticorrelated

## Abstract

In humans, the presence of a neural mechanism triggered by anticorrelated random-dot stereograms have been theorized based on animal models from invasive studies, but have not been experimentally verified with the use of electroencephalography. In this study, we employed a phase-consistent, temporally modulated alternating depth stereogram stimulus, where we created anticorrelation by inverting the contrast between the eyes. We recorded the electrical response of the resulting brain oscillations of our four participants using EEG in both the correlated and anticorrelated conditions and whether they perceived depth movement. Our analysis found that the correlated stereograms elicited a strong coherency at the even harmonics of the depth alternation, and the anticorrelated stimulus created lower coherency peaks at the first harmonic of the depth alternation, even when participants did not report the depth movement to be visible. While both conditions created a diminishment of spectral power in the beta band, we found that the anticorrelated condition created increased spectral power in the alpha band. We experimentally verified the presence of a neural mechanism triggered by anticorrelated random-dot stereograms in the human brain with our coherency analysis and that it would not have been detected with the conventional spectral analysis due to the weakness of the response. We hypothesize that the decreased beta oscillations are related to either visual discomfort and visual attention to our stimulus, and that the increased alpha oscillations in the anticorrelated condition is a response to the incorrect depth information created by the stereogram.

## 1. Introduction

**Figure 1:**
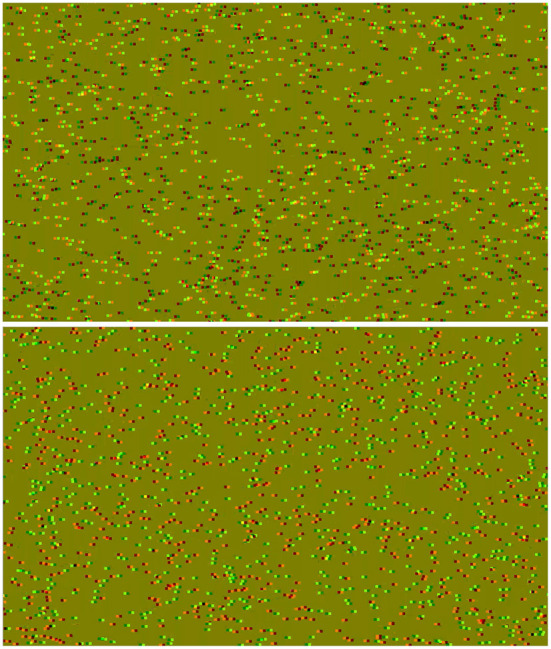
Anaglyph stereograms, rendered to be viewed with red-green glasses. Top: A correlated stereogram. Bottom: An anticorrelated stereogram. Both of these stereograms have the same horizontal bars encoded as depth information.

Stereopsis is a very unique function of human vision: while being non-essential for survival, it is one of the most computationally intensive features (Brown et al., 2003) that not only was discovered much later than other aspects of vision (Crone, 1992), but it also develops the latest in life (Giaschi et al., 2013). It is evolved to process binocular disparity information, largely independently of the luminance of the presented visual stimulus. A popular method to study this mechanism is with the use of Bela Julesz's random-dot stereograms (Julesz, 1971), where once the observer's visual system has solved the stereo correspondence problem, a simple pattern may be perceived (Scharstein and Szeliski, 2002). The dynamic correlated random-dot stereogram (dCRDS) can be rendered using a computer, by updating the locations of the dots in each subsequent frame. This type of stimulus works with humans (Lehmann and Julesz, 1978) and primates, and the underlying neural circuitry is well documented (Orban et al., 2006). Compared to the dCRDSs, the use of anticorrelated dynamic random-dot stereograms (dACRDS) where one eye's image has the contrast inverted, is a more subtle stimulus. The dACRDS does not create a sensation of depth (Hibbard et al., 2014) in the normal sense when the correlation alone is changed with the same depth information preserved. Figure 1 shows the same depth information (encoded as binocular disparity) rendered into a CRDS in the top and an ACRDS in the bottom, but none of the observers asked could identify the pattern shown in the ACRDS in the lower part of the figure. In order for a dACRDS to provide any sort of sensation of depth, special measures need to be taken: for example, for it to produce reversed depth at all, the dot density must be below 2% (Cumming et al., 1998); reversed depth perception can also occur when a dACRDS disk is shown surrounded by a dCRDS annulus in the central visual field (Aoki et al., 2017), but this reverse perception of depth also depends on the size of the gap between the two visual stimuli (Asher and Hibbard, 2018). Further, depth perception can be reversed when the temporal aspects of vision are exploited: when dACRDS mixed with zero-disparity dCRDS dot noise has been demonstrated to modulate (augment or degrade) depth perception, when it is flashed very briefly in the central visual field and the correlation turns during this short time (Zhaoping, 2021). Moving away toward the peripheral visual field where the spatial resolution drops considerably, dACRDS has also been demonstrated to elicit reversed depth perception when the stimulus is intentionally projected into this area (Zhaoping and Ackermann, 2018).

Neural response was detected for dACRDS visual stimuli using functional magnetic resonance imaging (Preston et al., 2008) in the visual cortex (V1, V3) and in the lateral occipital area in humans. In primates, it was detected in the primary visual cortex using extracellular single-unit recording (Cumming and Parker, 1997).

While the above studies have demonstrated inverse depth perception with special measures taken, comparatively few studies employed temporally modulated dACRDS visual stimuli, and even fewer conducted psychophysics studies using dACRDS as a depth movement stimulus.

In humans, the cortical response for binocular disparity has previously been studied with Electroencephalography (EEG). One particular approach for studying the neural mechanism that processes it is called frequency tagging: this technique uses a rendered stereogram that is temporally modulated, creating some sort of alternation of depth. Allowing for the event-related potential (ERP) generated at the onset of the visual stimulus to settle, the steady-state visual evoked potential (SSVEP) is analyzed further. In the SSVEP waveform, following a time-frequency transform, the temporal modulation frequency (or a harmonic of it) of the visual stimulus can be detected in the frequency domain. The visual stimulus can be a single frequency (Norcia and Tyler, 1984), or a combination of multiple frequencies (Skrandies and Jedynak, 1999). When multiple frequencies are used together, statistical analysis focuses on the beating (intermodulational) products of the stimulus frequencies (Baitch and Levi, 1988; Skrandies and Jedynak, 1999; Kamphuisen et al., 2008; Scherbaum et al., 2011; Norcia et al., 2015). For example in Baitch and Levi (1988), the absence of these intermodulational products was found to be correlated with “stereo blindness,” where the neural mechanism responsible for processing binocular disparity never developed due to untreated strabismus or amblyopia during childhood.

### 1.1. Signal processing in frequency-tagged EEG

It is important to note, however, that most of the above studies only analyzed their EEG waveforms in the time and frequency domains. In practice, they sampled the EEG waveforms using some sort of data acquisition device, and marked the timestamp of the trials for each channel. After they segmented their data knowing where the trials are in the continuous recording, they executed the fast Fourier transform (FFT) (Cooley and Tukey, 1965) on the segments for each channel. Then, they computed the spectrum from each channel's data. The magnitudes of the Fourier components at given frequencies is used to detect elevated spectral power, calculated with the use of various noise models and statistical methods.

Unfortunately, this spectral analysis method is not very sensitive. In order to detect the presence of a frequency-tagged signal in the spectrum, its spectral power must be higher than the noise power. The probability of successful detection of the signal is inversely proportional to the signal-to-noise ratio (SNR).

#### 1.1.1. Phase space and phase coherency: How the SNR is boosted at weak signals

Since all EEG signals are inherently very noisy, it is worthwhile considering how the noise is contaminating our signal. Some noise sources are easier to eliminate than others: we can do the EEG recording in a Faraday-cage, we could move away or turn off non-essential equipment to reduce the electrical noise at the input. Some noise is originating from the participant, and we can do something about it: we can tell the participant not to move the arms, not to wiggle the foot, or not to blink during the trials. Other participant-originated noise sources, such as the presence of cardiovascular pulses or irrelevant brain activity, are beyond our control. Eliminating these would be catastrophic to the participant and most probably to the experimenter as well.

**Figure 2:**
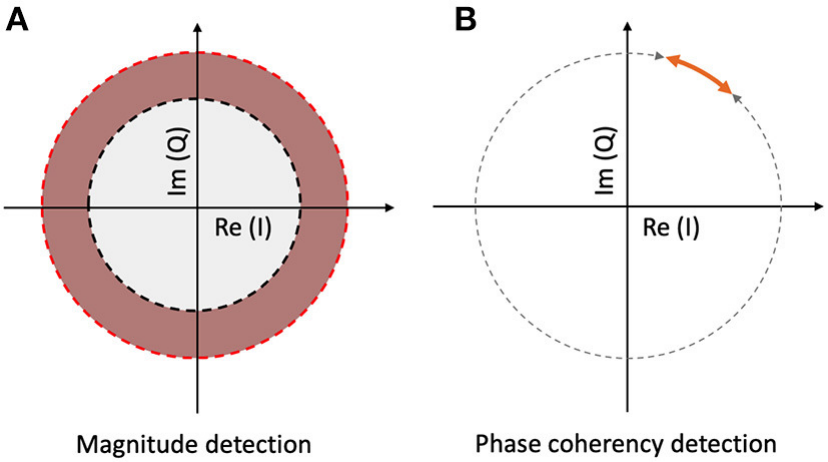
Signal detection methods with spectrum and coherency at a particular temporal frequency. **(A)** The spectral power is significantly elevated, when the vector reaches the red annulus. The noise threshold is set by the radius of the gray circle. **(B)** The signal is phase-locked to the stimulus, when the normalized vector's angle is inside the orange interval. The noise threshold is determined by the gray interval.

At this point, we should consider the noise problem from one step further away. When calculating the spectrum of the digitized EEG signal, the output of the FFT is a series of complex numbers, with every complex number representing a Fourier component corresponding to a temporal frequency. If we know the sampling rate, and the temporal frequency we are looking for, we can select the appropriate component and calculate the spectral power by taking the magnitude of the complex number. If it is higher than our noise threshold (see Figure 2A), then we detected our signal in the frequency domain. The vast majority of the frequency tagging studies cited above employ this method.

As complex numbers can be represented as vectors, the length of a chosen vector will determine the magnitude of the spectral power, and the argument (angle) of the vector will determine the phase of the component in question. At this point, we can split the noise phenomena described above to two parts: the amplitude noise (1) which changes the vector's length; and the phase noise (2) which changes the direction of the vector.

During the calculation of the spectrum both of these noise sources are preserved, and they decrease the SNR, which in turn reduces the probability of successful detection.

However, under the assumption that the temporal frequency of the stimulus is constant and the signal propagation time inside the brain is (near) constant across the trials, we can just keep the phase angle of the vector, and ignore its amplitude. When grouping several trials together, for example by segmenting the trials and aligning them to stimulus onset, the vectors at a temporal frequency that is phase-locked to the stimulus due to an oscillation triggered by it will have similar phase angles throughout. Additionally, if we take the confidence interval of the phase angles across the trials instead of the phase angle values themselves (this is shown in Figure 2B), and express them as a fraction of the full circle, we will have a single metric, the phase coherency, which tells us which frequency components will be more likely phase-locked and thus related to the stimulus. A phase coherency of 0 means that the phase of the frequency in question is effectively random across trials, and a phase coherency of 1 means that the frequency in question is always in the exact same phase across trials. This method not only rejects the amplitude noise component, but also diminishes the phase noise component as well, thereby making this technique far (more than 4 times) more sensitive than the spectral evaluation.

The first frequency tagging application of this technique was done by Norcia and Tyler (1984), with parts of the signal processing being done using discrete analog components.

In addition to being more sensitive, there are two additional benefits of the coherency analysis: an artificially generated data set is perfectly suitable for control trials (1), because in phase space, the noise is always uniformly distributed by principle. Any signal that is not phase-locked to the stimulus will have a uniformly distributed random phase at each trial (and a coherency value of 0 or at least a low value), irrespective of what type of process generated it. Therefore, for the purpose of the analysis, a simple white noise distribution can be used, provided that the number of trials matches the number of trials in the experimental data set. Any signal that will have a direct relationship with the stimulus will have a greater coherency value than what one would get from the artificially generated data. However, this should be used with caution, because this technique fails to detect incoherent oscillations caused by the stimulus. Luckily, it is easy to create the spectrogram with continuous wavelet transforms (Daubechies, 1990), as they are readily available in common EEG analysis packages, such as EEGLAB (Delorme and Makeig, 2004).

Furthermore, to improve the chance of detecting the signal, the recorded trials from several participants may be pooled together at the trial level to make a large data set (2): unlike with the spectral power or time-domain analysis, there is no risk of one powerful outlier driving the mean values of the distributions, because the phase coherency analysis effectively ignores the magnitudes of the Fourier components. In phase space, every vector carries the weight of 1, because the vectors are essentially normalized. However, due to the different signal propagation times for each individual (Norcia and Tyler, 1984), pooling the data together at the trial level carries the risk of having lower coherency values. Provided that the stimulus frequency is low enough, the probability of detecting the signal can actually be improved due to the lower noise threshold created by the higher number of trials, making this controversial type of data pooling a worthwhile trade-off in this particular application.

The temporal waveform of the stimulus may be sinusoidal, which will result in a single frequency after computing the spectrum. However, if a non-sinusoidal periodic waveform is used as a stimulus, the spectrum will contain several harmonics, which we can turn to our advantage: in this case, if the phase of the base harmonic of the stimulus is constant across trials, so will be the phases of all the subsequent harmonics as well. If a special type of square wave is used, where the two half-periods are identical in length (as in, the signal having a “50% duty cycle”), the signal will only contain the odd harmonics, with the magnitudes of the harmonics strictly monotonically decreasing for every subsequent harmonic. This way, a single temporal frequency is enough for a frequency tagging study, as it is possible to detect the presence of the harmonics themselves. With the added redundancy of this method, we can investigate the maximum bandwidth for a stimulus response, or estimate the SNR based on how many harmonics were found, or even find out whether any non-linear operation happens that may be linked to processing as the stimulus signal travels toward the EEG set through the participant's brain.

Another key difference between coherency and spectrum from a frequency tagging study point of view is that the coherency describes the closeness of the relationship between the neural response and the stimulus. A spectral peak, unlike a coherency peak, may be less likely related to the stimulus, and special measures need to be taken to eliminate false positives.

All the above, together with an example code is discussed in greater detail in Derzsi (2021), where two different coherency formulae are given for different situations. An explained mathematical proof that describes the relationship between common processing techniques, the SNR and the probability of successful detection is given in the Appendix of Derzsi (2017) and Derzsi (2020), with all of these being based on the detection probability of a multi-channel phase shift-keyed signal in communications engineering (Proakis and Salehi, 2008). Additionally, also in Derzsi (2021), a separate formula is given to calculate the number of trials required for a given SNR, which may be useful in estimating how many trials will be needed for a weak signal.

In this paper, we combine the use of temporally modulated dCRDS, dACRDS with human electroencephalography (EEG). We created a frequency tagging experiment similar in spirit to Norcia and Tyler (1984) study, but with anticorrelated stereograms. We aim to find out whether we will be able to detect phase-locked activity in frequency tagged dACRDS in humans, and compare how the signals relate to the conscious detection of binocular disparity-defined depth movement.

## 2. Methods

**Figure 3:**
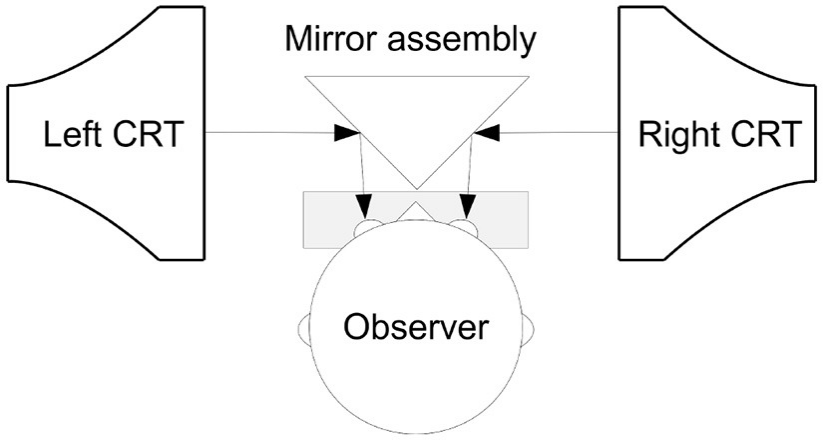
The stereoscopic display hardware made with computer monitors and mirrors. The participant sits in front of this assembly with the electrode cap attached to the head. As the position of the head is critical with respect the display apparatus, the participant had the head placed on the chin rest in front of the mirror.

We created a 3D display from two identical Dell P992 monitors that were built into a Wheatstone-stereoscope set-up using commercially available mirrors and 3D-printed parts. A schematic of this arrangement is shown in Figure 3. To the participant, the fused image of the monitors was 45 cm away, and covered 40-by-30 degrees visual angle. The monitors were driven at a refresh rate of 100 Hz, and the random dots were rendered with Psychtoolbox (Brainard, 1997; Pelli, 1997; Kleiner et al., 2007) at 100 frames per second. Each dot was seen under a 0.05 by 0.05 degree angle. The stereogram was rendered with a 50% gray background. Fifty percent of the rendered dots were black and 50% of the dots were white. The dot density was 0.16%, and the mean luminance output was 57.5 *cd*/*m*^2^.

We recorded EEG from 4 adult participants (2 males, 2 females, age 23.5 ± 3.5 years) who had normal or corrected to normal vision and verified to be able to perceive depth from binocular disparity, in short, 5–6 s trials. The randomly interleaved two conditions were dCRDS and dACRDS that showed a single alternating plane at 2.1 Hz, with identical (0.05°) positive and negative disparity values. The middle of the screen had nonius lines and a fixation cross that were always rendered with zero disparity.

Using EGI's 128-channel Geodesic Sensor Net (Electrical Geodesics, Inc, Eugene, OR, USA), we collected a total of 534 good trial recordings using dCRDS, and a total of 540 good trial recordings from dACRDS. The signal has been preprocessed using EGI's proprietary software, Net Station. We analyzed the waveforms for a single channel in the medial occipital area that is nearest to the visual cortex.

### Experimental design

The execution of the experiment was built up from 320 short, 5–6 s trials. To minimize the effect of fatigue, the participants started each trial, and they could have a break as often as they saw fit. Once a trial was started, a zero disparity dCRDS was presented for a randomized time between 1 and 1.5 s.

**Figure 4:**
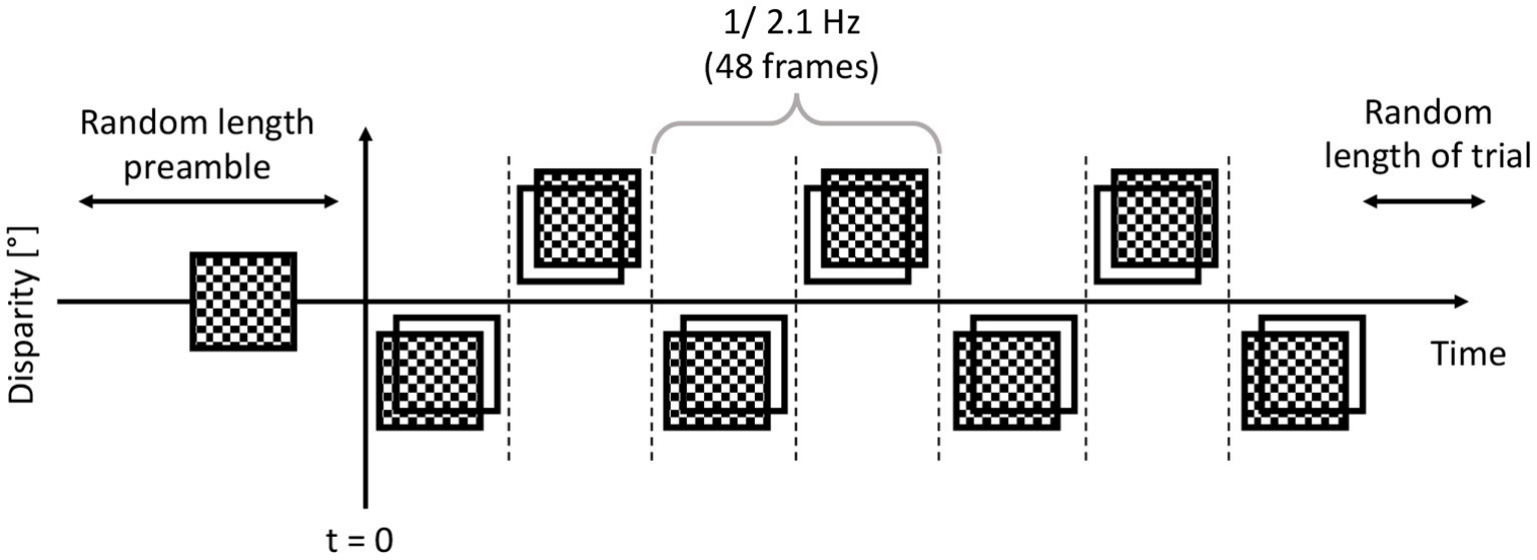
After the participant started the trial, before *t* = 0, the trial had zero disparity random dots displayed for 1...1.5 seconds. Then at *t* = 0, the dots had their disparity applied, exactly in the same frequency and phase in each trial. This alternating depth plane was displayed for a further 5-6 seconds.

Then, immediately afterwards, either a dCRDS or a dACRDS was presented for a randomized time between 5 and 6 s. The temporal modulation of the disparity plane was a 50% duty cycle square wave, and the timing pattern is shown in Figure 4. The participants were instructed to look at the screen, stay still, not to blink and not to move the eyes or any muscle for the duration of the trials. At the end of each trial, the participants were asked whether there was depth movement detected perpendicular to the screen plane in the stereogram presented in the trial.

The dCRDS presented was an alternating uniform depth plane of +/− 0.05 degrees, and the alternation frequency was 2.1 Hz, or at every 48 frames. The dACRDS had the same parameters, but the contrast was inverted on one screen. The examples of a disparity grating for both types of RDS are rendered in Figure 1.

### 2.1. EEG hardware and signal pre-processing

The 128-channel electrode cap we used in the experiment has silver-chloride electrodes, and each of them is coupled to the participant using sponges that were soaked in a saline-based electrolyte. All channels were kept below the impedance of 50 kΩ. Electrically, the system measured the voltage between a reference electrode at the apex and each channel separately, and the system operated at a sampling rate of 1 kHz. The timing of the stimulus was annotated using a photodiode attached to a dedicated area on the screen which was invisible to the participant. After recording, the EEG signal was band-pass filtered between 0.1 and 70 Hz, and a notch filter (48–52 Hz) was applied to reduce interference from the 50 Hz mains hum. Then the continuous recording was segmented using the timing data of the onset of the stereogram with disparity within the trials. This way, the temporal depth alternation was exactly the same frequency and in the same phase across all the trials. If a trial had an eye blink or eye movement artifact or more than 13 channels were noisy or it was marked as a bad trial during recording for any reason, it was excluded from further analysis. There was a time limit of 60 min per recording session for each participant.

### 2.2. Data processing and analysis

After exporting the preprocessed data from the EEG software, we processed and analyzed the data further using our own script in Matlab and with EEGLAB (Makeig et al., 1996). In each recorded good trial, we took the Fast-Fourier Transform of 5 s of the EEG signal from the moment the alternating grating had appeared on the screen, and we calculated the inter-trial coherency (Norcia and Tyler, 1984; Derzsi, 2021) across trials for the spectrum between 1 and 15 Hz.

We also imported the data to EEGLAB, and generated the time-frequency transforms of trials, using the built-in continuous wavelet transform (Daubechies, 1990) method. We also used EEGLAB's built-in statistical functions for the time-frequency transform.

### 2.3. Statistics

For each condition, we analyzed the inter-trial coherency of the signal of a single channel measured at the medial occipital area at the first six harmonics (2.1, 4.2, 6.3, 8.4, 10.5, and 12.6 Hz) of the depth alternation frequency of the stereogram. We compared the inter-trial coherency distributions of the EEG trials against the noise threshold line. This line was generated using a process similar to bootstrapping in statistics: we created 10,000 synthetic data sets containing white noise for the same number of trials as our data, calculated the coherency values, and took the 95th percentile of the resulting distribution for every frequency. While the probabilities can be computed precisely using the ratio of how many coherency values are above the noise threshold and how many were used in the analysis, as a rule of thumb, if a coherency value is above the noise threshold at a harmonic of the stimulus frequency, it has a significance level of smaller than 0.05.

In EEGLAB, we used the built-in statistical thresholding on the time-frequency transform. The threshold was also set to be the same probability as our analysis, 0.05.

## 3. Results

### 3.1. Psychophysics

**Figure 5:**
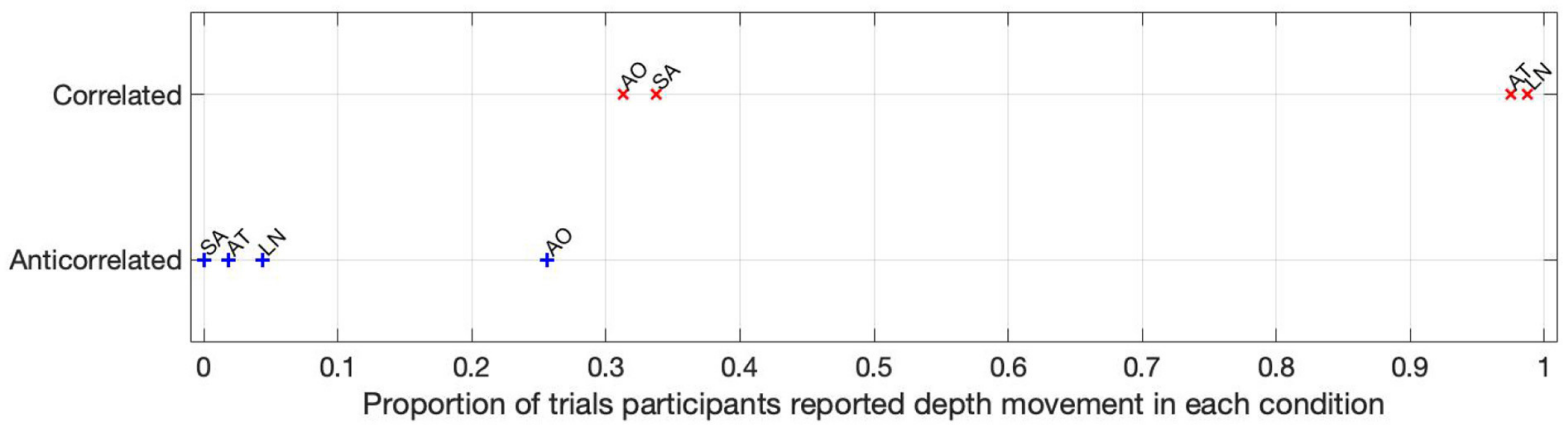
The depth movement perception ratio for each participant in each condition. The labels next to the data points are individual participant codes.

The proportion of trials where depth movement was detected is shown in Figure 5. Participants AT and LN detected the depth movement in almost every (97.5 and 98.75%, respectively) trial in the correlated condition, but did not see depth movement in almost any (1.8 and 4.37%, respectively) trial. Participant SA saw some depth movement in the correlated condition, but it is below chance (33.75%), and reported absolutely no depth movement in the anticorrelated condition at all. Participant AO also reported depth movement below chance (31.25%) in the correlated condition, and similarly this participant reported the highest proportion of depth movement, but still below chance (25.62%) in the anticorrelated condition.

### 3.2. Coherency of the harmonics of the stimulus alternation frequency

**Figure 6:**
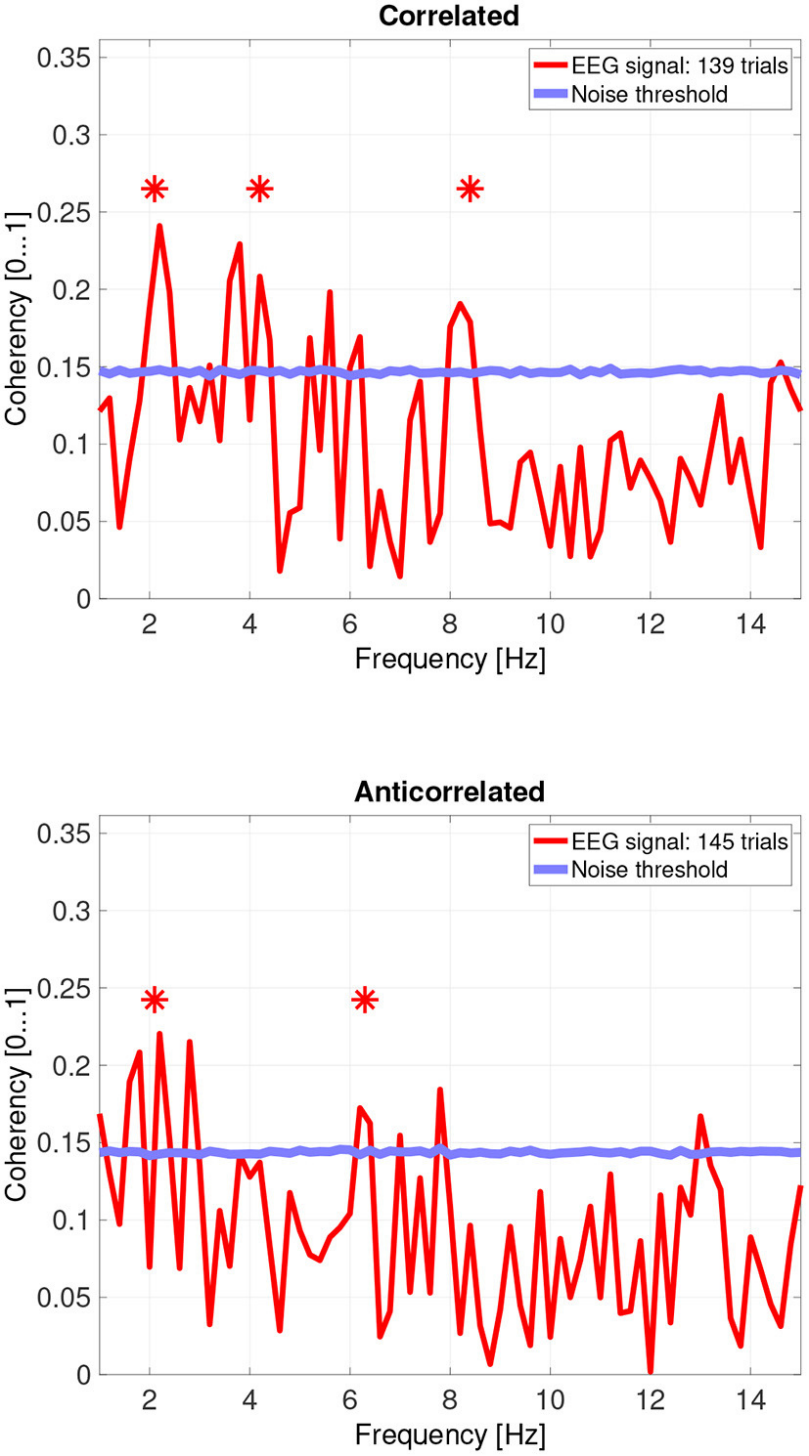
Coherency values from participant SA. The stars above the peaks indicate successful detection of the harmonic of the stimulus frequency. Note that the coherency plots are in phase space, and that they are not showing spectrum: a higher coherency value imply a stronger relationship to the stimulus. The “Noise threshold” is created with 10,000 data sets having the same number of trials, with the trials containing only white noise. The line shows the 95*th* percentile of the coherency values created from these synthetic data sets.

**Figure 7:**
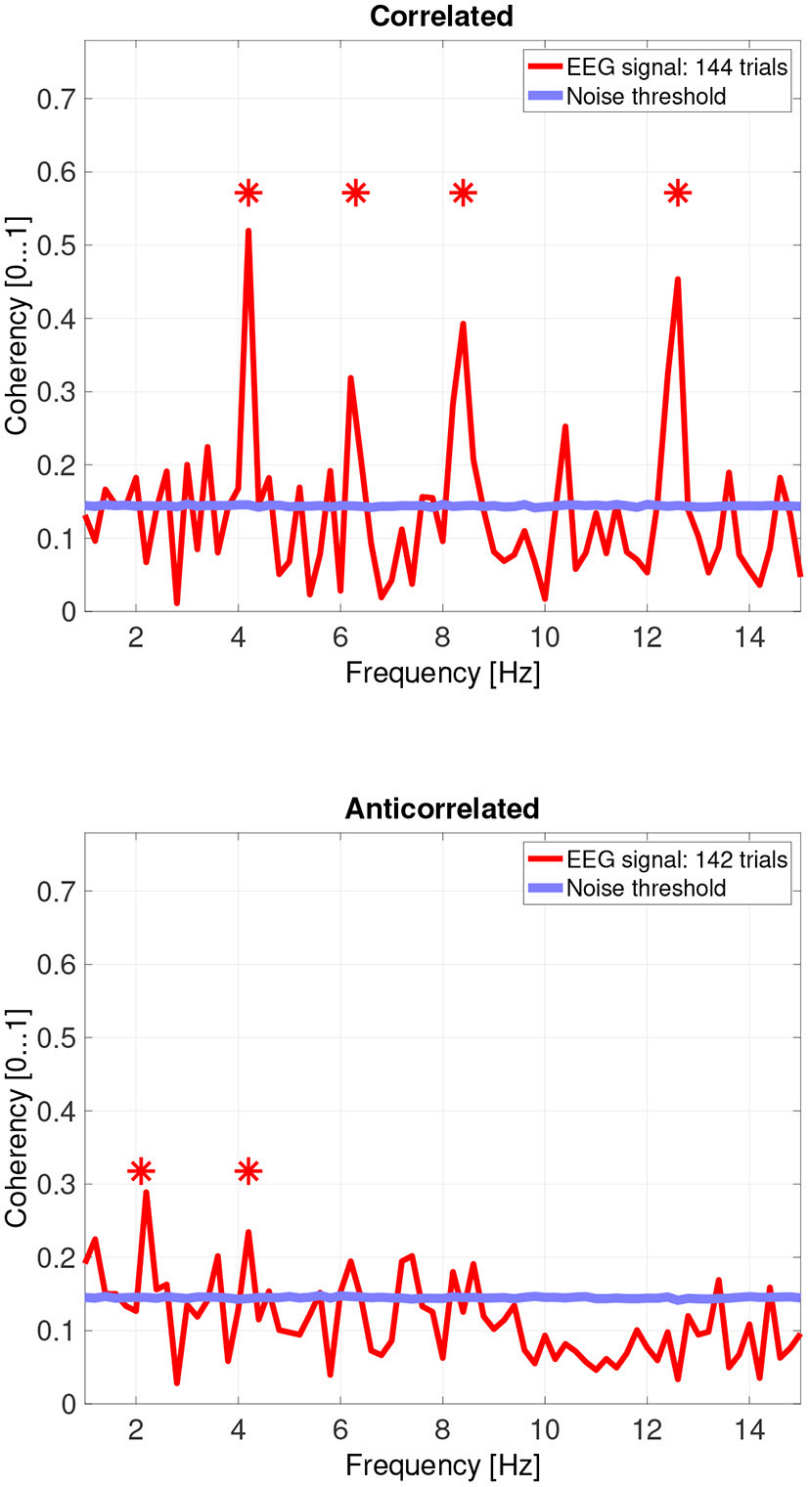
Coherency values from participant LN. The stars above the peaks indicate successful detection of the harmonic of the stimulus frequency. Note that the coherency plots are in phase space, and that they are not showing spectrum: a higher coherency value imply a stronger relationship to the stimulus. The “Noise threshold” is created with 10,000 data sets having the same number of trials, with the trials containing only white noise. The line shows the 95*th* percentile of the coherency values created from these synthetic data sets.

**Figure 8:**
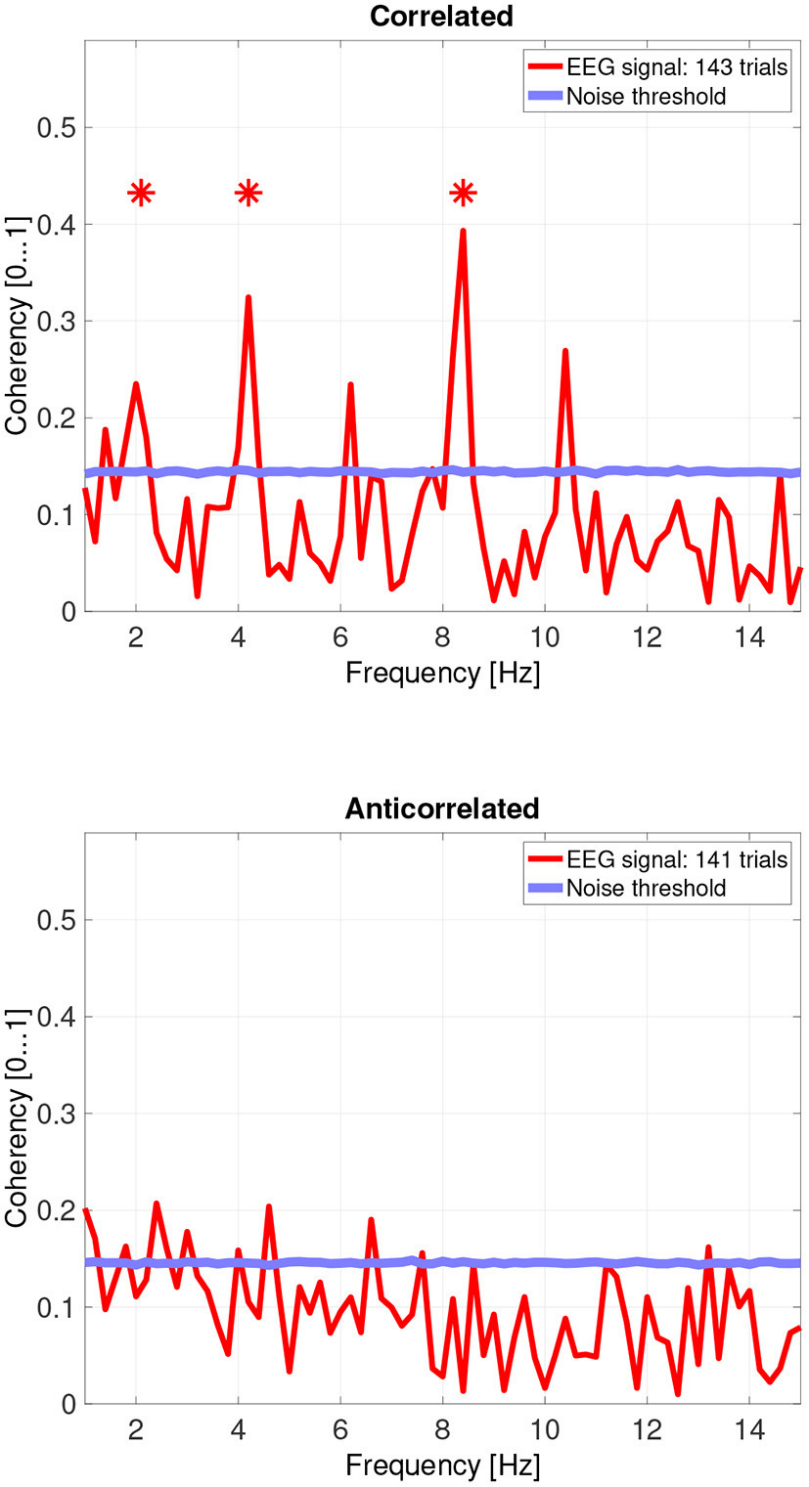
Coherency values from participant AT. The stars above the peaks indicate successful detection of the harmonic of the stimulus frequency. Note that the coherency plots are in phase space, and that they are not showing spectrum: a higher coherency value imply a stronger relationship to the stimulus. The “Noise threshold” is created with 10,000 data sets having the same number of trials, with the trials containing only white noise. The line shows the 95*th* percentile of the coherency values created from these synthetic data sets.

**Figure 9:**
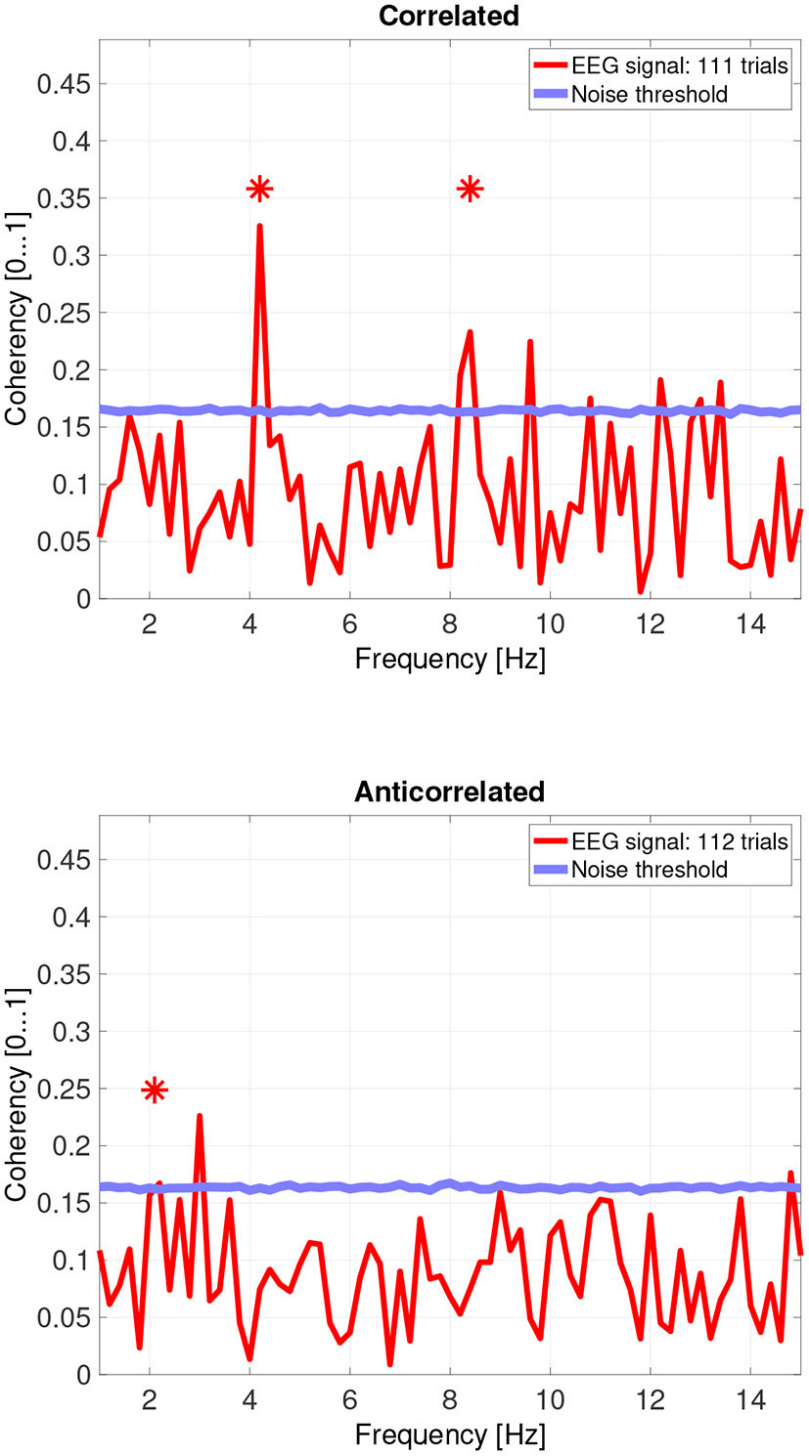
Coherency values from participant AO. The stars above the peaks indicate successful detection of the harmonic of the stimulus frequency. Note that the coherency plots are in phase space, and that they are not showing spectrum: a higher coherency value imply a stronger relationship to the stimulus. The “Noise threshold” is created with 10,000 data sets having the same number of trials, with the trials containing only white noise. The line shows the 95*th* percentile of the coherency values created from these synthetic data sets.

While there are individual variations, we detected the second and fourth harmonics of the depth alternation frequency for all participants in the correlated condition. The coherency values at these frequencies range between 0.2 and 0.5. For the participants who are more sensitive (see the top plots in Figure 6 for participant SA and Figure 7 for participant LN) the coherency values are higher, and more harmonics are detected than for our less sensitive participants (top plots of Figure 8 for participant AT and Figure 9 for participant AO).

**Figure 10:**
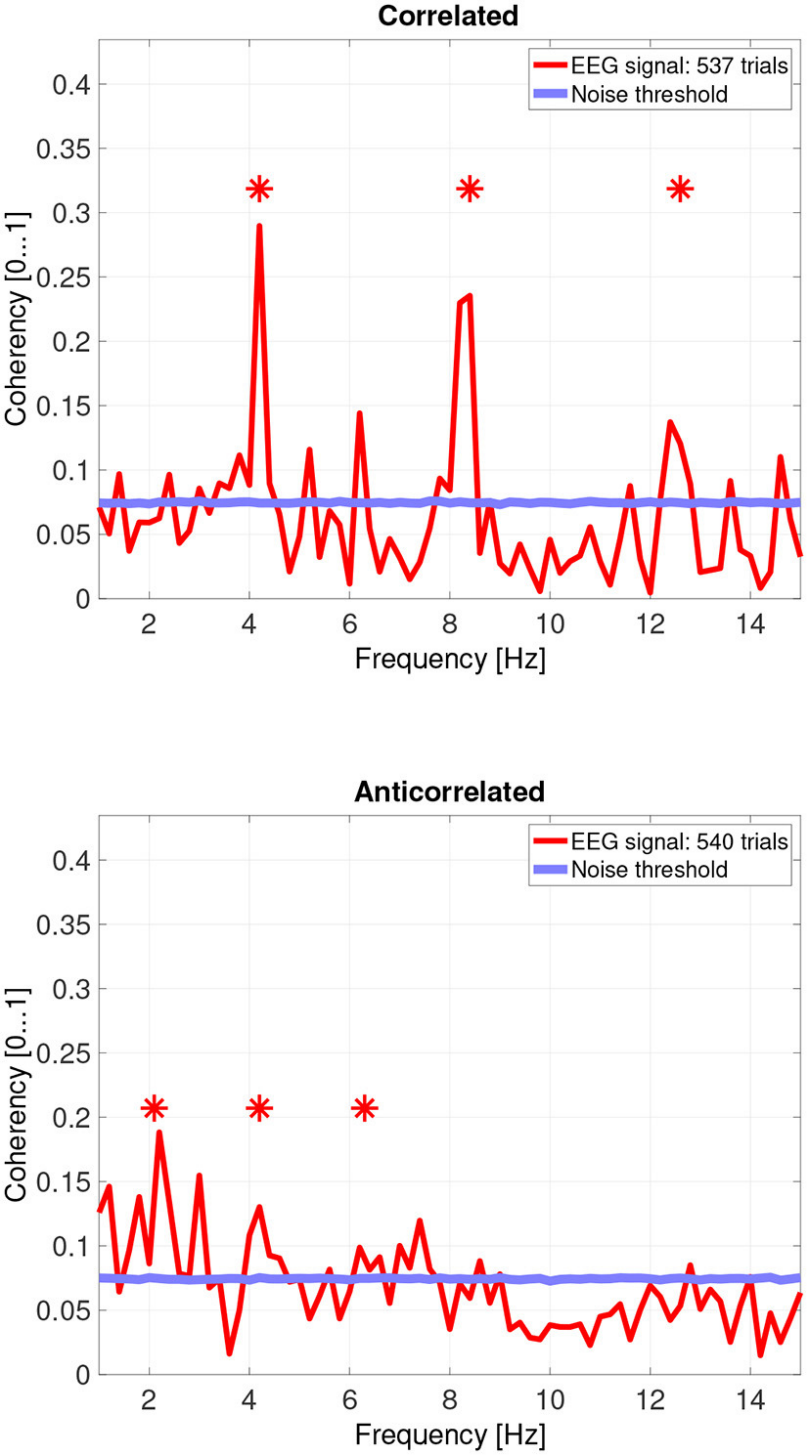
Coherency values of the pooled trials from four participants. The stars above the peaks indicate successful detection of the harmonic of the stimulus frequency. Note that the coherency plots are in phase space, and that they are not showing spectrum: a higher coherency value imply a stronger relationship to the stimulus. The “Noise threshold” is created with 10,000 data sets having the same number of trials, with the trials containing only white noise. The line shows the 95*th* percentile of the coherency values created from these synthetic data sets.

When pooling the data together at a trial level (Figure 10, top plot), the second, fourth and sixth harmonics are significantly elevated. Despite the overall coherency values being lower, the coherency peaks look more marked, because the noise threshold fell from approximately 0.15 to 0.08.

In the anticorrelated condition, while the noise threshold is nearly identical due to the similar number of trials being used, the coherency values are marginally lower than for the correlated condition. For the more sensitive participants (in the bottom plots of Figure 6 for participant SA and Figure 7 for participant LN), the first harmonic is detected in both cases, and one higher harmonic has a weak coherency value. From the participants with worse SNR, we either could not detect the signal at all (Figure 8, bottom plot), or it was borderline (Figure 9, bottom plot).

While the pooled data (see Figure 10's bottom plot) also show a set of reduced coherency values, the presence of the first, second and third harmonics of the depth alternating frequency is being detected. There is also a band of markedly lower coherency values, between 9 and 11 Hz.

Interestingly, there seem to be other frequencies, especially in the anticorrelated condition (around 3 Hz, 7 Hz, and others in the pooled data) where the calculated coherency values are above the level of the noise distribution which are close to, but not at the harmonics of the signal.

### 3.3. Time-frequency transform of the trials

**Figure 11:**
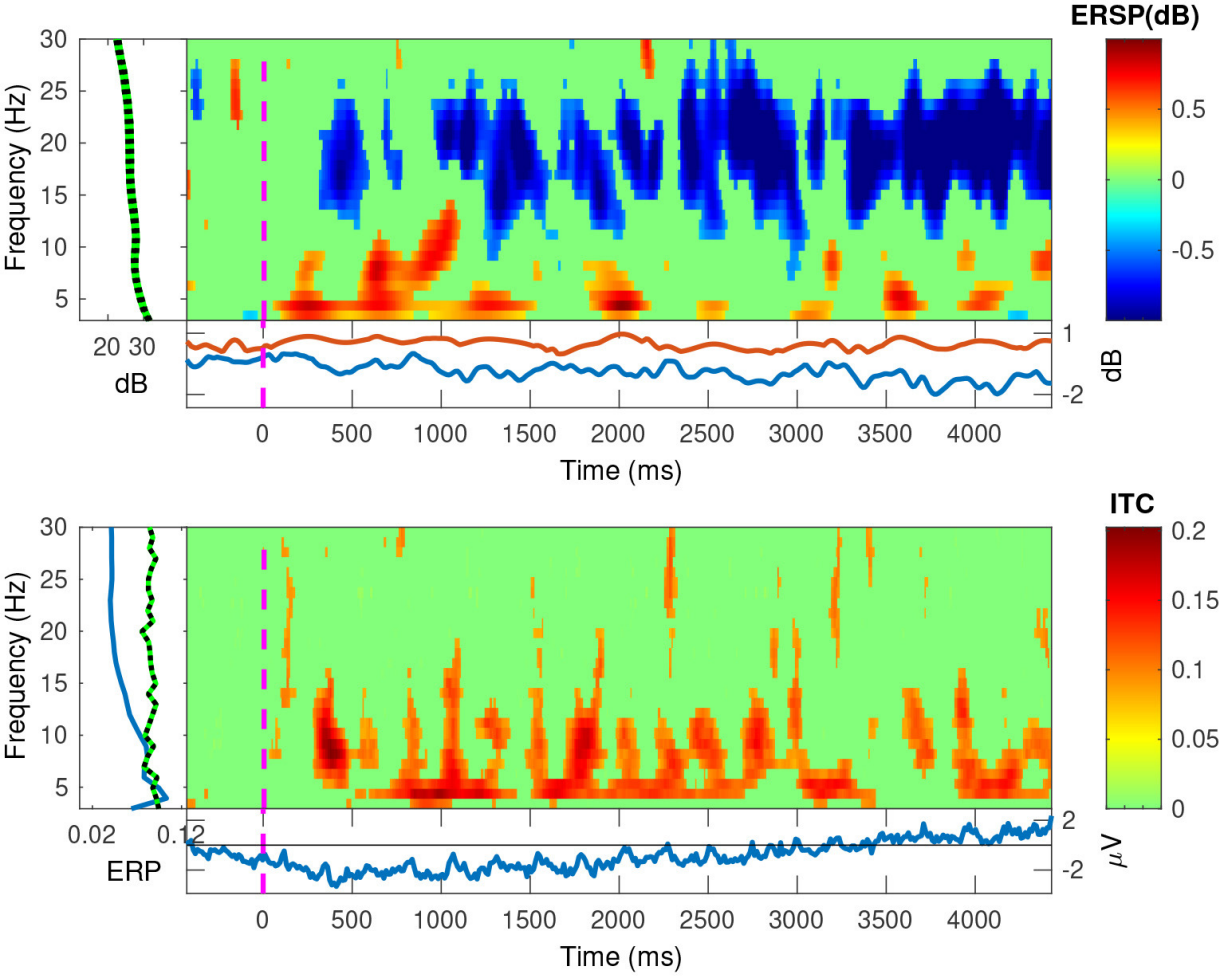
The EEGLAB output for our pooled data, for the correlated condition: The ESRP plots show relative spectral power density changes as a function of time, and the ITC plots show the coherency values as a function of time. The green area is a mask to hide statistically insignificant data. The band at the low frequencies in both plots show a strong neural response at the second and fourth harmonics of the stimulus. The ESRP plot also shows a marked decrease in the beta (13–30 Hz) band.

**Figure 12:**
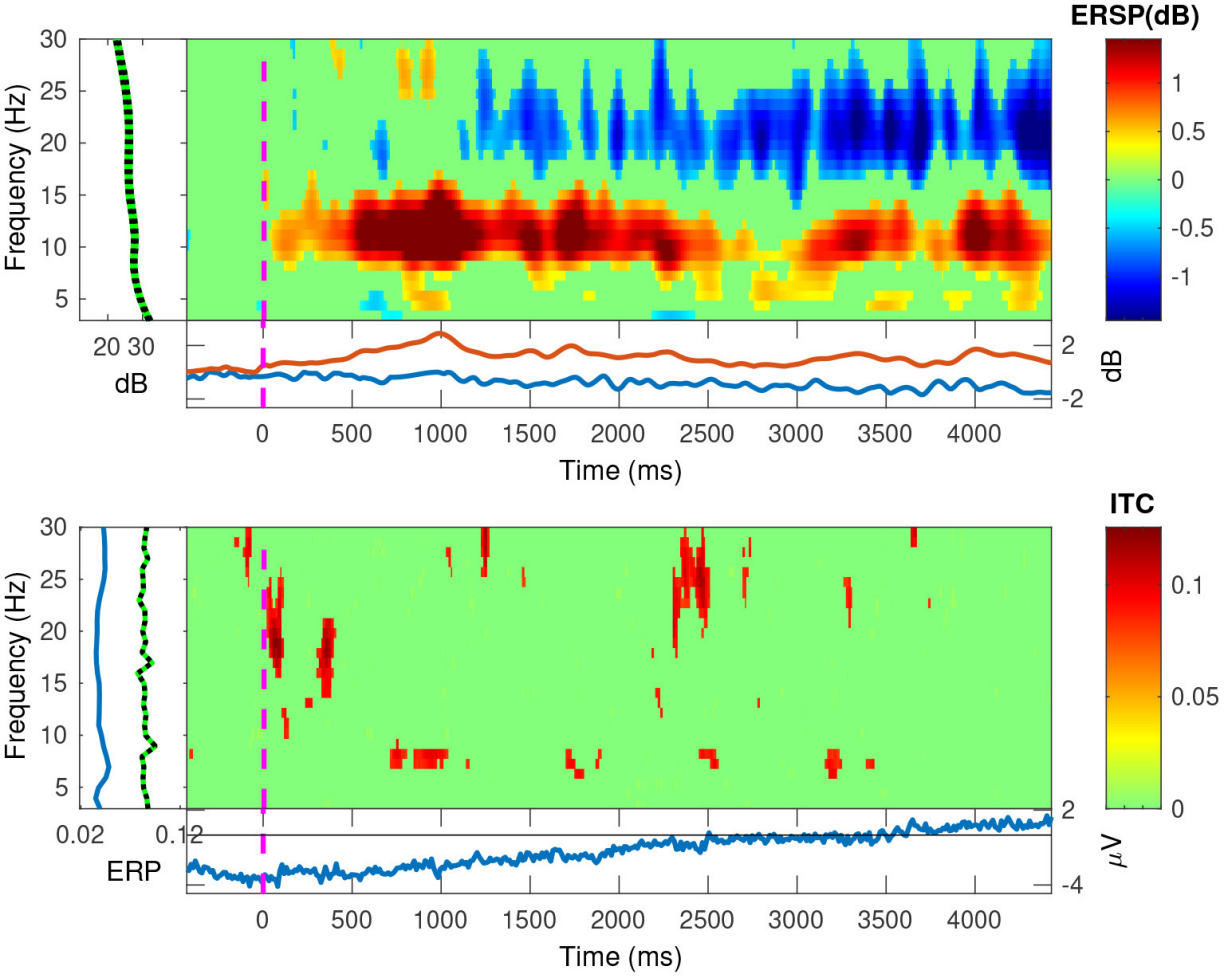
The EEGLAB output for our pooled data, for the anticorrelated condition: The ESRP plots show relative spectral power density changes as a function of time, and the ITC plots show the coherency values as a function of time. The green area is a mask to hide statistically insignificant data. While the ESRP plot shows the marked decrease of spectral power in the beta (13–30 Hz) band, it also shows a marked increase of spectral power in the alpha (8–12 Hz) band. As ITC plot shows nothing significant, these oscillations do not appear to be phase-locked to our visual stimulus.

In Figures 11, 12, we plotted the event-related spectral perturbations (ERSPs) in the top plots, and the inter-trial coherency (ITC) in the bottom plots for our data, pooled at a trial level. Additionally, by setting EEGLAB's bootstrapping method, we masked the statistically insignificant parts in the plots. In both conditions, we see a slight, but significant, 1 dB decrease of spectral power in the 13–30 Hz (beta) band. For the anticorrelated condition (Figure 12, top plot), there is also a similarly small, but also significant, 1 dB elevation in the 8–12 Hz (alpha) band.

Further, in the correlated condition of the pooled data (see Figure 11), we see that the ERSPs in the temporal frequencies of 2 and 4 Hz increased, which corresponds with the elevated inter-trial coherency at the second and fourth harmonics of the depth alternation frequency of the stimulus. Interestingly, unlike with our analysis method, EEGLAB failed to detect the coherency of the second harmonic of the stimulus in the anticorrelated condition (Figure 12, bottom plot).

## 4. Discussion

### 4.1. Coherency analysis

There is a strong coherency measured in the correlated condition. Since this stimulus is very similar to what was in Norcia and Tyler's (1984) study, we expected even stronger coherency values (0.8–1) at the second harmonic of the signal. In our analysis, we detected generally lower coherency values, but at more harmonics. We attribute this difference to the way the data was processed. Norcia and Tyler used discrete filters on the actual signal itself for the second harmonic of the stimulus before recording, whereas we extracted the same data using our own signal processing techniques. Since our recording bandwidth was much higher, we found reduced coherency values for several even harmonics for the correlated condition. Since the disparity modulation was essentially a 50% duty cycle square wave, we know that spectrum of the modulated disparity stimulus signal only contained odd harmonics. Therefore, we suggest that the coherency values measured at the even harmonics are the result of temporally modulated activity in the human visual system, and the odd harmonics present in the EEG recordings are either some intermodulational products, or simply the frequency tagged signal passing through the human visual system.

The effect of pooling is also visible: for example, the mean coherency value of the second harmonic is 0.34 across our participants, and a mean noise threshold of 0.15 yields an SNR of about 2.6 in the coherency data for the correlated condition. While pooling across participants reduced the coherency value at this harmonic from 0.34 to 0.29, we have observed a considerable reduction of the noise threshold, from 0.15 to 0.08. This yields a coherency SNR of 3.6, which is almost a 40% increase. In this case, pooling across trials was a worthwhile trade-off especially for the anticorrelated condition, where we were only able to detect a harmonic in just 3 of the 4 participants. Most probably we would have been able to detect the signal in all our participants, if we had more trials in the experiment. However, this was not possible due to participant fatigue, and due to hardware limitations such as the saline-based electrolyte drying out in the electrode cap. Additionally, the anticorrelated condition's frequency tagged signal is extremely weak, which makes the coherency values at the harmonics very low (shown in Figure 10's bottom plot), it would have been impossible to detect the frequency tagged signal with the traditional spectral analysis with the same number of trials. The absence of any relevant harmonics in the time-frequency plots in Figure 12 further confirms this.

### 4.2. Signal-to-noise ratio types

Calculating the SNR directly from the coherency data is unfair: the EEG signal has been previously filtered; trials marked as “bad” were selectively rejected; and only a part of each trial was used in the analysis where we knew that the frequency-tagged signal was presented. All these measures improve the chance of detecting the signal, and calculating the SNR after these steps gives a far more optimistic value than what it actually is in the raw recording.

A much fairer comparison is to estimate the SNR in the time domain using the minimal number of trials required for detection, as it includes every possible noise source the EEG set can pick up. Based on our data, we can approximate that around only 60–80 trials are required to detect the second harmonic in the correlated condition, and around 120–150 trials are required to detect the signal in the anticorrelated condition. Therefore, we can estimate (Derzsi, 2021) that the time-domain SNR is around 0.5 (the signal is about half the power of noise) for the correlated condition, and around 0.1 (the signal is ten times below the noise) for the anticorrelated condition at the second harmonic of the stimulus frequency. For contrast, spectral evaluation even with taking the phase angles into account, would need approximately 100 trials for the correlated, and around 500 trials for the anticorrelated condition to achieve a 0.05 significance level. The SNR values are far smaller for every subsequent harmonic, as the Fourier-transform of the depth alternation waveform has strictly monotonically decreasing amplitudes for subsequent harmonics. These weak harmonics potentially require thousands of trials to be successfully detected with the conventional spectral analysis, and would be practically impossible to achieve with a single participant in the same experiment.

### 4.3. “Birdie” signals

In engineering, the term “birdie” refers to an unrelated, parasitic oscillation in the system. In our EEG study, the signals with lower coherency peaks between the harmonics that are above the noise threshold are considered birdies. These signals may have originated from a separate phase-locked oscillation related to our stimulus or occurred by chance. We indeed observed similar peaks at the synthetic noise trials as well but with much lower magnitudes. We speculate that the presence of these peaks between the two harmonics, especially in the pooled data, is either the signal passing through partially processed by the human visual system, or is being generated by the means of intermodulation originated from the non-linear nature of the medium the signal is traveling through. Some other peaks are more difficult to explain: in Figure 10, we can see these peaks at 6.2 Hz in the correlated condition and at 3 Hz in the anticorrelated condition. We hypothesize that some of these non-harmonic “birdie” frequencies are either intermodulational products, or evidence for some sort of synchronized oscillation in the human visual system originated from a processing mechanism we do not yet understand. Analyzing the data using wavelet transforms with EEGLAB (see the ERSP plot in Figure 12) clearly shows a significant level of oscillations in the alpha (8–12 Hz) band emerging from time zero onwards in the anticorrelated condition. This may suggest that the oscillation, while being incoherent, is related to the presented stimulus. Perhaps we are measuring the two sides of the same system: a series of incoherent oscillations that may contain some coherent components. Interestingly, the alpha band has one of the lowest coherency values in both plots of Figure 10, which further suggests that its presence might be the result of the visual stimulus.

### 4.4. ERSPs and EEGLAB's coherency

In the correlated condition, we can see that the second (4 Hz) and fourth (8 Hz) harmonic of the signal's ERSP is significantly increased. Based on this, along with the increased coherency values, we suggest that this is due to the disparity alternation in the stimulus, i.e., the harmonics of the “tagged” frequency. In the anticorrelated condition, the time-frequency analysis shows a significantly increased ERSP in the alpha (8–12 Hz) band. However, it is important to consider that EEGLAB's plotting function uses a relatively broad, 1 Hz frequency resolution, even if it uses continuous wavelet transform instead of FFT. For comparison, our coherency analysis used a higher, 0.2 Hz frequency resolution. Since the harmonics themselves are very precisely set in frequency, using a lower frequency resolution will result in the lowering of the spectral (and through the spectrum, the coherency) peaks, and this is the reason that EEGLAB does not show several harmonics in the time-frequency transform of the pooled data to be significantly elevated in the correlated condition, and it shows no significant results at all for the harmonics in the anticorrelated condition. However, the two results of the two independent analysis methods are similar, and it does not change our findings.

### 4.5. Differences between reported depth movement and signal detection in the EEG recordings

Based on the psychophysics data shown in Figure 5 and the principles of frequency tagged EEG processing, one would imply that there is a proportionality between the likelihood of depth movement perception and the strength of coherency. This is not the case. Participant LN and participant AT almost exclusively saw depth movement in the correlated condition. While we successfully detected the frequency tagged signal in LN's recordings in the anticorrelated condition, they were practically absent in AT's data. We believe this is due to a challenge every EEG experimenter has to face day-by-day: AT had considerably longer hair than LN, which made it more difficult to maintain a low electrode impedance throughout the experiment, which in turn reduced the SNR in the recording. When comparing the peak coherency values of the second harmonic between LN's (see Figure 7 top plot at 4.2 Hz: 0.54) and AT's (see Figure 8 top plot at 4.2 Hz: 0.33) data at the second harmonic, we can see that AT's values are marginally smaller, but the noise threshold is around the same, approximately 0.15. We imply that this relatively small drop in the effective SNR made the signal undetectable in the anticorrelated condition. Had we obtained more trials with participant AT, there would have been a good chance that the signal would have been detected in the anticorrelated condition as well.

Participant SA worked differently: this participant did not detect depth movement above chance in the correlated condition, and unlike any other participant, did not detect depth movement in the anticorrelated condition at all. At the same time, SA's coherency plots (see Figure 6) clearly show that the signal was detected in both cases, implying that SA's visual system did indeed process the frequency tagged signal. We believe that this participant has been overly cautious when giving depth perception responses, and this would explain the lower proportion.

Then, there is participant AO. This participant responded with the lowest proportion at the correlated condition, and the highest score at the anticorrelated condition. Both of them, below chance. Participant AO's coherency responses were weak (see Figure 9, top plot at 4.2 Hz: 0.33) at the second harmonic's frequency in the correlated condition, and shows it to be barely significant (see Figure 9, bottom plot, at 2.1 Hz: 0.155) at the base harmonic of the temporal modulation frequency in the anticorrelated condition. We believe that this participant did not pay due attention to the experiment and at the task at hand, and the psychophysics responses were below chance due to the general lack of enthusiasm from the participant's side. At the same time, the coherency data clearly shows that the signal did make the way through the visual system, although with considerably more attenuation than with every other participant.

### 4.6. Broader implications

While the correlated condition essentially replicates the findings of Norcia and Tyler (1984), the choice of the waveform enabled us to estimate the maximum temporal bandwidth of human stereopsis. It is considerably slower than the flicker fusion threshold (Landis, 1954) or luminance-based apparent motion detection thresholds (Tyler, 1973; Anstis et al., 2000), but due to the complexity of stereopsis in the human visual system, this is not a surprise. For the correlated condition, where the sixth harmonic (12.6 Hz - period time: 79.4 ms) was detected would imply that the peak disparity angular velocity where neural response could be detected is 1.26 degrees per second. For the anticorrelated condition, where only the third harmonic (6.3 Hz–period time: 158.7 ms) was found, the peak disparity angular velocity where neural response could be detected is considerably lower, 0.63 degrees per second. In reality, these peak velocities will probably be higher, and will likely depend on the actual disparity magnitude as well - in a similar way to apparent motion.

Depth motion in temporally modulated dACRDS was generally not consciously detected by our participants. This is despite the low dot density and net luminance output constancy. Perceptually, both to the experimenter and the participants, dACRDS felt like it forces convergence on vision, potentially rendering the monitors at a small positive (far away) disparity, and there was no distinct feeling of depth pulsation one would get with the dCRDS. Sadly there was no vergence-capable eye tracker employed during the experiment, which is why this was not reported in the results.

Another interesting and unexpected finding is the presence of alpha oscillations in the anticorrelated condition. These oscillations are difficult to trace because they are not produced by a single source in the brain (Nunez et al., 2001), but they have been shown to be involved when optical illusions are presented (Lange et al., 2013, 2014). If we consider that a dACRDS as a type of stimulus that one should never see in a natural environment and that it triggers an inverse response in the disparity-sensitive neurons in the visual cortex, then one can infer that this erroneous triggering will result in the human visual system trying to restore the malformed signal it was supplied with. From this point of view, the dACRDS is in fact an optical illusion, because it can create a false (in some cases, inverted depth) perception. With this in mind, the presence of alpha oscillations may be considered as an indicator of an “error correction” process in response to the dACRDS: these oscillations have been shown to be related to an inhibitory process (Klimesch et al., 2011; Klimesch, 2012), which periodically resets the visual information processing pathway. In computer networks, this is a well-known feature (Tanenbaum, 2002), and is implemented in a large number of different protocols to prevent the collision of messages on the same channel or to prevent the hosts from falling out of sync with each other. With the incomplete information that the dACRDS carries, perhaps part of the human visual system is forced into the human stereopsis equivalent of “connection reset” state for the duration of the stimulus. However, even for the visual system, the same oscillation may represent simultaneous and opposite processes from different areas in the brain (Clayton et al., 2018), which will make further analysis of this phenomenon near-impossible. Perhaps, in a real life situation, this anomalous nature of the stimulus is one of the reasons why diamonds are considered to be so mesmerisingly shiny: the shape of the crystal can reflect light at very narrow angles, making it possible to project a beam into one eye, with no beam projected into the corresponding visual angle in the other eye. The effect is more powerful when the crystal is illuminated from a spot light and presented with a dark background, which essentially creates the anticorrelation.

Unlike with alpha oscillations, both conditions also seem to have created a diminishment of spectral power (see Figure 12, top plot) in the Beta band. Without other studies replicating this finding, the presence and reason for this oscillation remains to be unanswered. It may be related to visual discomfort (Cho et al., 2012), or visual attention (Wróbel, 2000; Kamphuisen et al., 2008). Since the visual stimulus in this experiment was rather more functional than aesthetic, it is impossible to tell from this data which played a more significant role. Anecdotally both the experimenter and all the participants reported the visual stimulus as boring, dull and generally numbing. Unfortunately this is unavoidable in order to create a visual stimulus which only carries a singular visual cue.

## 5. Conclusion

In this paper, we have shown that a uniform plane of binocular disparity presented in the form of a dynamic correlated random-dot stereogram can elicit a strong enough response in the human visual system, that even the sixth harmonic of the temporal modulation frequency can be detected with the EEG frequency-tagging method. When the temporally modulated dynamic anticorrelated stereogram is presented at the same temporal frequency, the response measured in the human visual system diminished greatly, but is still detectable when the inter-trial coherency of electroencephalogram is analyzed.

Based on the presence of coherent oscillations at the harmonics of the stimulus frequency, and the incoherent oscillations in the alpha band emerging from the onset of the stimulus, we suggest that we have detected a response for anticorrelated dynamic random-dot stereograms in the human visual system.

## Data availability statement

Data and the processing script are available upon request. Requests to access these datasets should be directed to zd8@nyu.edu; ha5dzs@gmail.com.

## Ethics statement

The studies involving human participants were reviewed and approved by Newcastle University-Institute of Neuroscience Ethics Committee. The patients/participants provided their written informed consent to participate in this study.

## Author contributions

ZD designed and constructed the experimental hardware, collected data, wrote the signal processing script, wrote the GPU script, and wrote the paper.

## Funding

Funding for the project was provided by the Wellcome Trust (Grant No. 099756/Z/12/Z). We also have received a high-performance graphics card from Nvidia.

## Conflict of interest

The author declares that the research was conducted in the absence of any commercial or financial relationships that could be construed as a potential conflict of interest.

## Publisher's note

All claims expressed in this article are solely those of the authors and do not necessarily represent those of their affiliated organizations, or those of the publisher, the editors and the reviewers. Any product that may be evaluated in this article, or claim that may be made by its manufacturer, is not guaranteed or endorsed by the publisher.
